# Mapping of genomic regions associated with arsenic toxicity stress in a backcross breeding populations of rice (*Oryza sativa* L*.*)

**DOI:** 10.1186/s12284-019-0321-y

**Published:** 2019-08-09

**Authors:** Varunseelan Murugaiyan, Jauhar Ali, Anumalla Mahender, Umair M. Aslam, Zilhas Ahmed Jewel, Yunlong Pang, Corinne M. Marfori-Nazarea, Lin-Bo Wu, Michael Frei, Zhikang Li

**Affiliations:** 1 Rice Breeding Platform, International Rice Research Institute (IRRI), 4031 Los Baños, Laguna Philippines; 2 Plant Nutrition, Institute of Crop Sciences and Resource Conservation (INRES), University of Bonn, D-53012 Bonn, Germany; 3 State Key Laboratory of Crop Biology, College of Agronomy, Shandong Agricultural University, Taian, 271018 People’s Republic of China; 4 National Key Facility for Crop Gene Resources and Genetic Improvement, Institute of Crop Science, Chinese Academy of Agricultural Sciences, Beijing, 100081 People’s Republic of China

**Keywords:** Rice, Arsenic toxicity, Single nucleotide polymorphisms, Quantitative trait loci, Candidate genes

## Abstract

**Background:**

Arsenic (As) is an unwanted toxic mineral that threatens the major rice-growing regions in the world, especially in South Asia. Rice production in Bangladesh and India depends on As-contaminated groundwater sources for irrigating paddy fields, resulting in elevated amounts of As in the topsoil. Arsenic accumulating in rice plants has a significant negative effect on human and animal health. Here, we present a quantitative trait locus (QTL) mapping study to identify candidate genes conferring As toxicity tolerance and accumulation in rice (*Oryza sativa* L*.*) seedlings. An early backcross breeding population consisting of 194 lines derived from a cross between WTR1 (*indica)* and Hao-an-nong *(japonica*) was grown in hydroponics for 25 days, from the seventh day exposed to an environmentally relevant concentration of 10 ppm As.

**Results:**

Arsenic toxicity leads to significantly negative plant responses, including reduced biomass, stunted plant growth, reduced leaf chlorophyll content, and increased shoot As concentration ranging from 9 to 20 mg kg^− 1^. Marker-trait association was determined for seven As-related traits using 704 single nucleotide polymorphism (SNP) markers generated from a 6 K SNP-array. One QTL was mapped on chromosome 1 for relative chlorophyll content, two QTLs for As content in roots were mapped on chromosome 8, and six QTLs for As content in shoots were mapped on chromosomes 2, 5, 6, and 9. Using the whole-genome sequence of the parents, we narrowed down the number of candidate genes associated with the QTL intervals based on the existence of a non-synonymous mutation in genes between the parental lines. Also, by using publicly available gene expression profiles for As stress, we further narrowed down the number of candidate genes in the QTL intervals by comparing the expression profiles of genes under As stress and control conditions. Twenty-five genes showing transcription regulation were considered as candidate gene nominees for As toxicity-related traits.

**Conclusions:**

Our study provides insight into the genetic basis of As tolerance and uptake in the early seedling stage of rice. Comparing our findings with the previously reported QTLs for As toxicity stress in rice, we identified some novel and co-localized QTLs associated with As stress. Also, the mapped QTLs harbor gene models of known function associated with stress responses, metal homeostasis, and transporter activity in rice. Overall, our findings will assist breeders with initial marker information to develop suitable varieties for As-contaminated ecosystems.

**Electronic supplementary material:**

The online version of this article (10.1186/s12284-019-0321-y) contains supplementary material, which is available to authorized users.

## Background

Rice (*Oryza sativa.* L) is one of the world’s most important staple crops, consumed by more than half of the world’s population, and it plays a major role in the entry of mineral nutrients into the food chain (Global Rice Science Partnership 2013; Yorobe et al. 2016). Traditionally, rice is grown in flooded paddy fields, which can harbor unwanted toxic heavy metals such as arsenic, cadmium, mercury, and lead, which represents a threat to human and cattle health (Norton et al. 2010; Pandey et al. 2015). Compared with the other major cereals, rice takes up large amounts of arsenic (As), a highly toxic class I carcinogenic metalloid, which naturally occurs worldwide in the environment and is widely distributed in Earth’s crust (Liu et al. 2012a). Rivers originating from the greater Himalayas carry As from their rock sediments to the densely populated rice-growing plains of South and Southeast Asia, making the major rice-growing belt of Asia vulnerable to As contamination (Sohn 2014; Zhou et al. 2017). In addition, climate change is threatening rice production in these areas. With frequent occurrence of drought and saltwater intrusion in the Ganges-Brahmaputra deltas of India and Bangladesh, farmers depend on groundwater for irrigation of their crops (Guan et al. 2010; Marcaida et al. 2014; Wang et al. 2015). This groundwater is an additional source of As discharged from the naturally As-rich aquifers (Nickson et al. 1998). Rice production in the major regions of Bangladesh and India uses As-contaminated groundwater for irrigation. Typically, 4 to 8 mg kg^− 1^ of As occurs in flooded paddy soil, but the concentration can reach 83 mg kg^− 1^ in some parts of Bangladesh and West Bengal regions of India (Zhang et al. 2008; Norton et al. 2010).

Rapidly growing populations need more food despite the increased labor cost, reduced farmland, and water scarcity that threaten rice production. Long-term use of contaminated groundwater for irrigating crops has resulted in a significant increase in As in the topsoil, thereby contaminating the food chain (Pandey et al. 2015). Governments of developing Asian nations are encouraging farmers to adopt modern technologies such as direct-seeded rice (DSR) and alternate wetting-drying (AWD) as a cost-efficient sustainable method to reduce the environmental footprint of rice production (Fischer et al. 2007; Singh Chauhan et al. 2015). Toxic levels of As in the topsoil can potentially affect the performance of DSR by hindering the most sensitive rice germination process and early seed establishment of the rice growth cycle (Abedin and Meharg 2002; Shri et al. 2009). Water management using AWD was proposed as one of the strategies to control As bioavailability in the soil-plant system (Mitra et al. 2017). However, rice varieties suitable for the AWD system under As-toxic soil have not been developed and tested in field conditions for implementation (Suriyagoda et al. 2018). Therefore, it is necessary to reveal the genetic basis of As tolerance and accumulation in the early seedling stage of rice, which will help rice breeders to develop As-tolerant varieties suitable for modern rice production technologies (Tuli et al. 2010).

As toxicity in rice plants triggers various symptoms, including low germination rate, poor seed establishment, reduced photosynthetic rates, stunted plant growth, low biomass production, sterility-related yield loss, and a physiological disorder called straighthead disease. These symptoms are often confounded with other soil-related problems associated with rice (Abedin et al. 2002a; Azizur Rahman et al. 2008). The toxic effects of As in rice vary upon thier chemical form, with the inorganic As species being more toxic than the organic species (Abedin et al. 2002b; Tripathi et al. 2013). The inorganic species of As found in the rice-growing environment are arsenite (As^(III)^) and arsenate (As^(V)^). In anaerobic flooded soil conditions such as submerged paddy fields, the reduced form As^(III)^ dominates and in aerobic soil conditions such as upland paddy fields its oxidized form As^(V)^ dominates (Tripathi et al. 2013; Pandey et al. 2015). Both forms are toxic to rice plants (Batista et al. 2011). Rice plants do not posses naturally evolved As transporters (Pandey et al. 2015), instead, As competes with chemically similar essential minerals to enter the plant system (Stoeppler 2003). As^(III)^ is physiochemically similar to the essential mineral silicon (Si) and thus competes with the Si uptake pathway. Alternatively, A^(V)^ is physicochemically similar to essential mineral phosphorous (P) and uses P acquisition pathways to enter the root system, and for efflux toward the xylem and various tissues (Clemens 2006; Zhao et al. 2009; Yang et al. 2018). Most rice genotypes possess a mechanism to retain much of the toxic As burden in the roots. However, a genotype-dependent proportion of As is translocated into the shoots and other tissues, including grains of the rice plant (Carey et al. 2010; Pandey et al. 2015). Since 35–55% of rice is produced in irrigated conditions (Ali et al. 2018b), As^(III)^ contributes the major As species loaded into rice plants (Zhao et al. 2010b). As^(III)^, which exists as the neutral molecule As (OH)_3,_ enters rice root cells through nodulin 26-like intrinsic proteins (NIPs), belonging to the aquaporin family of major intrinsic proteins (MIPs), which are non-permeable to As^(V)^ (Ma et al. 2008; Zhao et al. 2009). Widely spread NIP aquaporins also mediate the transport of a range of neutral molecules, including ammonia, urea, boric acid, and silicic acid (Zhao et al. 2010a). The silicon transporter in rice root *OsLSI1* has been suggested as the main As^(III)^ uptake protein, while As^(III)^ efflux from rice root cells to the xylem takes place through *OsLSI2* silicon-mediated transporter (Ma et al. 2008; Zhao et al. 2010b). However, none of the above genes associated with the As^(III)^ and As^(V)^ tolerance-low uptake genes were functionally characterized to use in breeding programs for the development of As-safe varieties.

Mapping QTLs to identify causative genes in populations is essential for trait improvement in breeding programs (Würschum 2012). Using backcross breeding populations has proven to be an effective strategy to dissect the genetic factors that underly the phenotypic complexity of nutrient-related traits with a simultaneous focus on varietal development (Xue et al. 2006; Ali et al. 2013; Wu et al. 2014). Although As interactions with rice have been well documented over the past two decades, only a limited number of QTL studies and genes associated with As have been reported for the development of As-safe varieties (Dasgupta et al. 2004; Zhang et al. 2008; Chen et al. 2013; Zhang et al. 2014). For As^(V)^, *AsTol* on chromosome 6 was the first QTL reported in rice for root tolerance and it is located close to a phosphate uptake QTL (Dasgupta et al. 2004). For As^(III)^, the first QTLs reported in rice are located on chromosomes 2 and 3 for As content in shoot and root, respectively, and chromosome 6 and 8 for As content in brown rice (Zhang et al. 2008). In this current study, as a component of the ongoing Green Super Rice (GSR) backcross breeding program at the International Rice Research Institute (IRRI) for the development of multi-tolerance rice varieties for the resource-poor farmers of Asia and Africa (Li and Ali 2017), we try to understand the genetic and molecular mechanisms underlying As tolerance and accumulation in rice seedlings to accelerate the development of As-tolerant and -safe varieties for contaminated ecosystems. The objective of the study has been the following: 1) to screen for As toxicity tolerance in early backcross breeding populations, 2) to identify QTLs related to As tolerance and accumulation using high-density SNP markers, and 3) to identify candidate genes for As-related traits using the whole-genome sequence approach.

## Results

### Performance of parental lines and BRILs

**Fig. 1:**
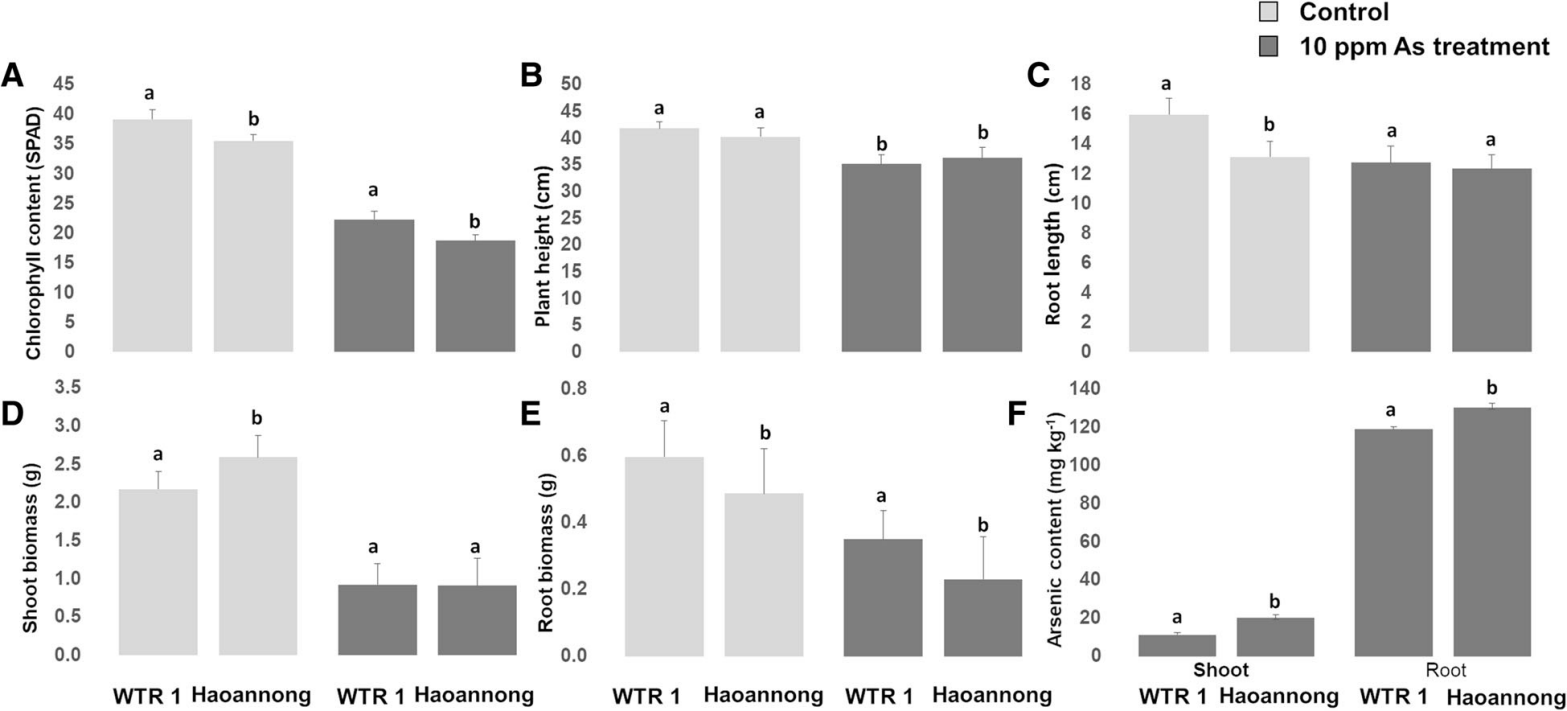
Phenotypic trait variation in parents of mapping population under As toxicity stress for 18 days. (A) Chlorophyll content; (B) plant height; (C) root length; (D) shoot dry weight; (E) root dry weight; (F) arsenic content in shoot and root tissue. Vertical bars represent standard errors of means (*n* = 3). Different letters above the data points indicate significant differences between genotypes by LSD test (*P* < 0.05)

**Fig. 2:**
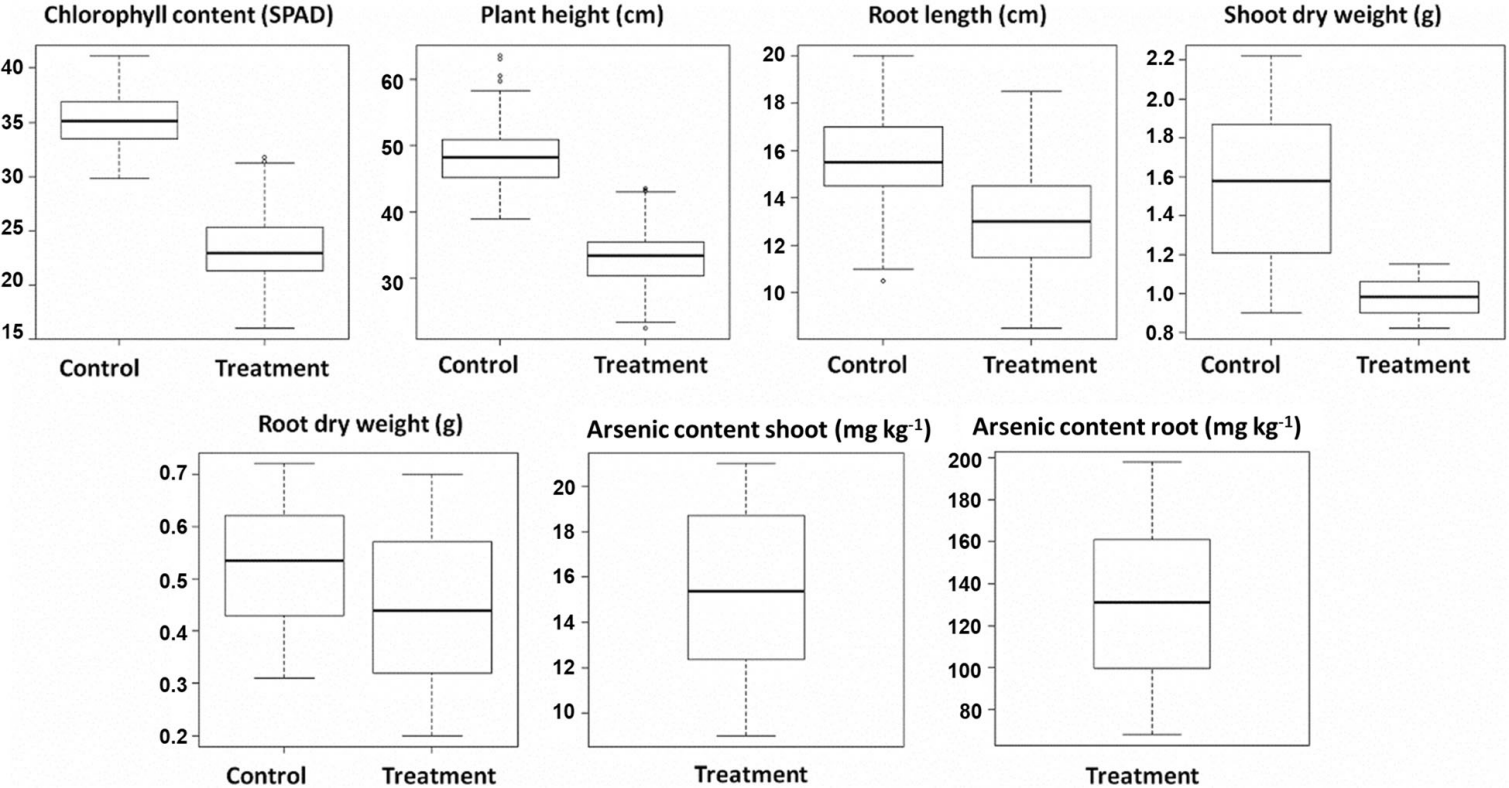
Box plot distribution of measured As-related stress in the population; control with no As and treatment with 10 ppm As a substitution for 18 days. Control: without As substitution, Treatment: with 10 ppm As substitution

Exposure to 10 ppm of As for 18 days induced a significantly negative response in parents and the mapping population. WTR1 showed more tolerance than Hao-an-nong in terms of chlorophyll content and biomass accumulation. For root length and plant height, Hao-an-nong showed higher tolerance. The parents WTR1 and Hao-an-nong showed highly significant differences in accumulating As between shoot and root tissue (Fig. 1). When averaged over all backcross recombinant inbred lines (BRILs), the mean values of shoot dry weight, plant height, and chlorophyll content decreased considerably in the As stress, except for root biomass and root length (Table 1). As content in BRILs ranged from 9.10 mg kg^− 1^ (AsG-195) to 20.99 mg kg^− 1^ (AsG-227) and 68.62 mg kg^− 1^ (AsG-185) to 197.10 mg kg^− 1^ (AsG-226) in shoot and root, respectively. Compared with the parents, 15 BRILs showed the lowest uptake of As concentration (≤10.00 mg kg^− 1^ in shoots and ≤ 80.00 mg kg^− 1^ in roots). In particular, two genotypes, AsG-187 (*GSR IR2–1-R14-N2-N5-N21-N42*) and AsG-189 (*GSR IR2–1-Y4-N4-N2-N3-N2*), showed better performances in low uptake of As content in roots and shoots (Additional file 1: Table S1). Most of the traits measured in the control and treatment conditions appeared to be normally distributed (Fig. 2). The overall results indicated that adequate genetic variability is present in the parental material and populations studied for QTL analysis.

**Table 1:** Descriptive statistics and ANOVA results for different phenotypes

Trait	Control			Treatment 10 ppm As			ANOVA result		
	Min	Max	Mean	Min	Max	Mean	G	T	G^*^T
Chlorophyll content (SPAD)	29.93	40.72	35.31	16.15	31.45	23.27	^***^	^***^	NS
Plant height (cm)	39.33	63.17	48.25	23.13	43.50	33.31	NS	^***^	^***^
Root length (cm)	10.83	19.83	15.86	9.00	18.17	13.00	^***^	^***^	^***^
Shoot dry weight (g)	0.91	2.22	1.56	0.82	1.15	0.98	^***^	^***^	^***^
Root dry weight (g)	0.31	0.72	0.53	0.20	0.70	0.45	^***^	^***^	NS
Arsenic content of shoots (mg kg^−1^)	ND	ND	ND	9.10	20.99	15.48	^***^	NA	NA
Arsenic content of roots (mg kg^− 1^)	ND	ND	ND	68.62	197.10	130.36	^***^	NA	NA

Significance levels are indicated at *P* < 0.001/^***^ Treatment and genotype treatment interaction effects were not analyzed for As content traits since in control conditions arsenic is not measured

*ND* not determined, *NA* not significant, *NA* not applicable, *G* genotype, *T* treatment

### Correlation among measured traits

**Fig. 3:**
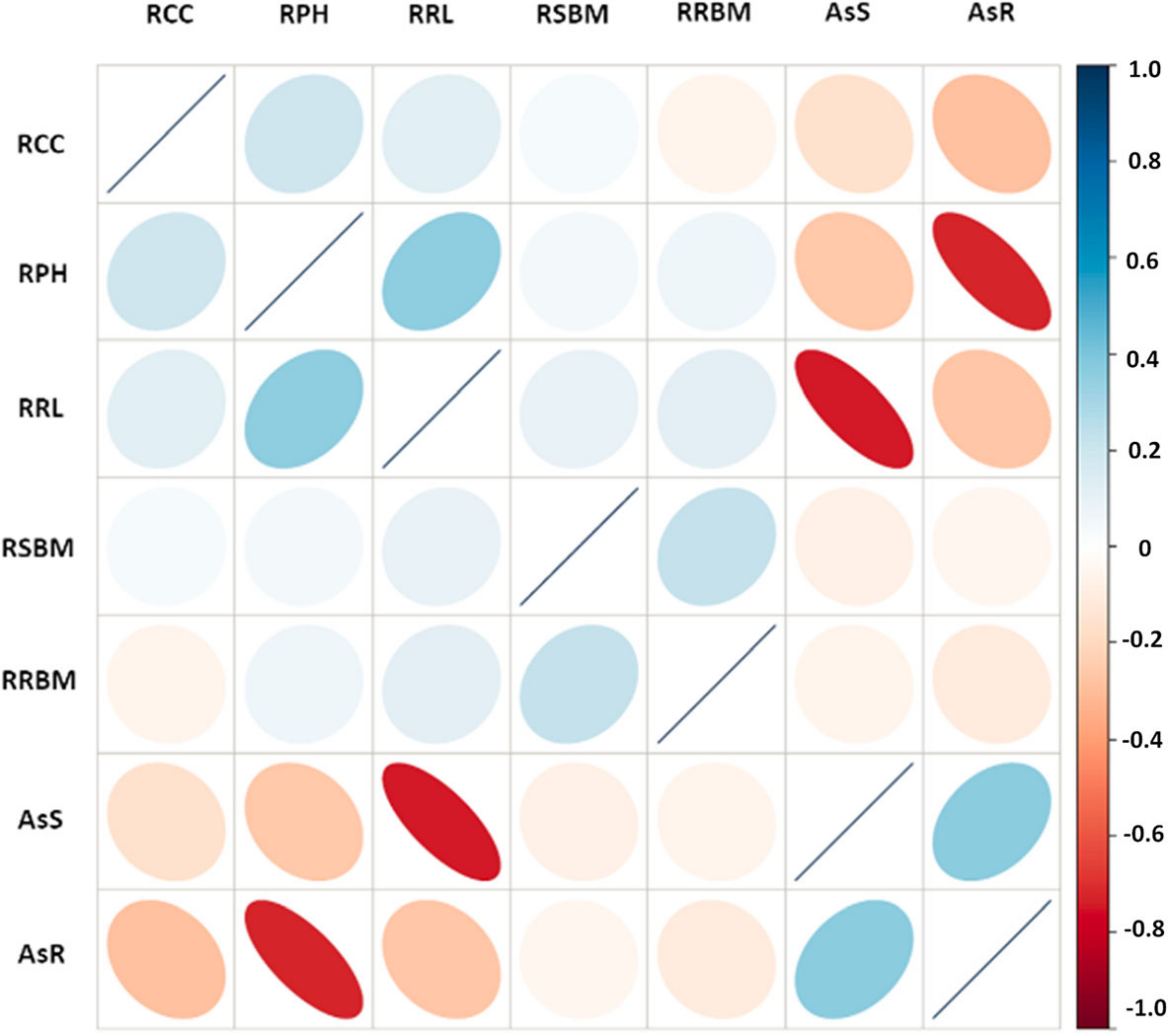
Heat map is showing the correlation coefficient of As-related traits. RCC: relative chlorophyll content; RPH: relative plant height; RRL: relative root length; RSBM: relative shoot biomass; RRBM: relative root biomass; AsS: As content in shoots; AsR: As content in roots

The study of correlation coefficient among various measured As-related traits is of great importance to predict the relationship between complex quantitative As content traits and simple quantitative morphological traits associated with As toxicity. For As content in shoots, the data showed a significantly negative correlation with relative plant height, chlorophyll content, and root length (Fig. 3). For As content in roots, there was a significant negative correlation with relative root length and plant height. A significant and positive correlation coefficient was recorded between As content in shoots and roots, relative root length and relative shoot length, and relative shoot biomass and relative root biomass.

### SNP markers generated by 6 K SNP-array for QTL mapping

**Fig. 4:**
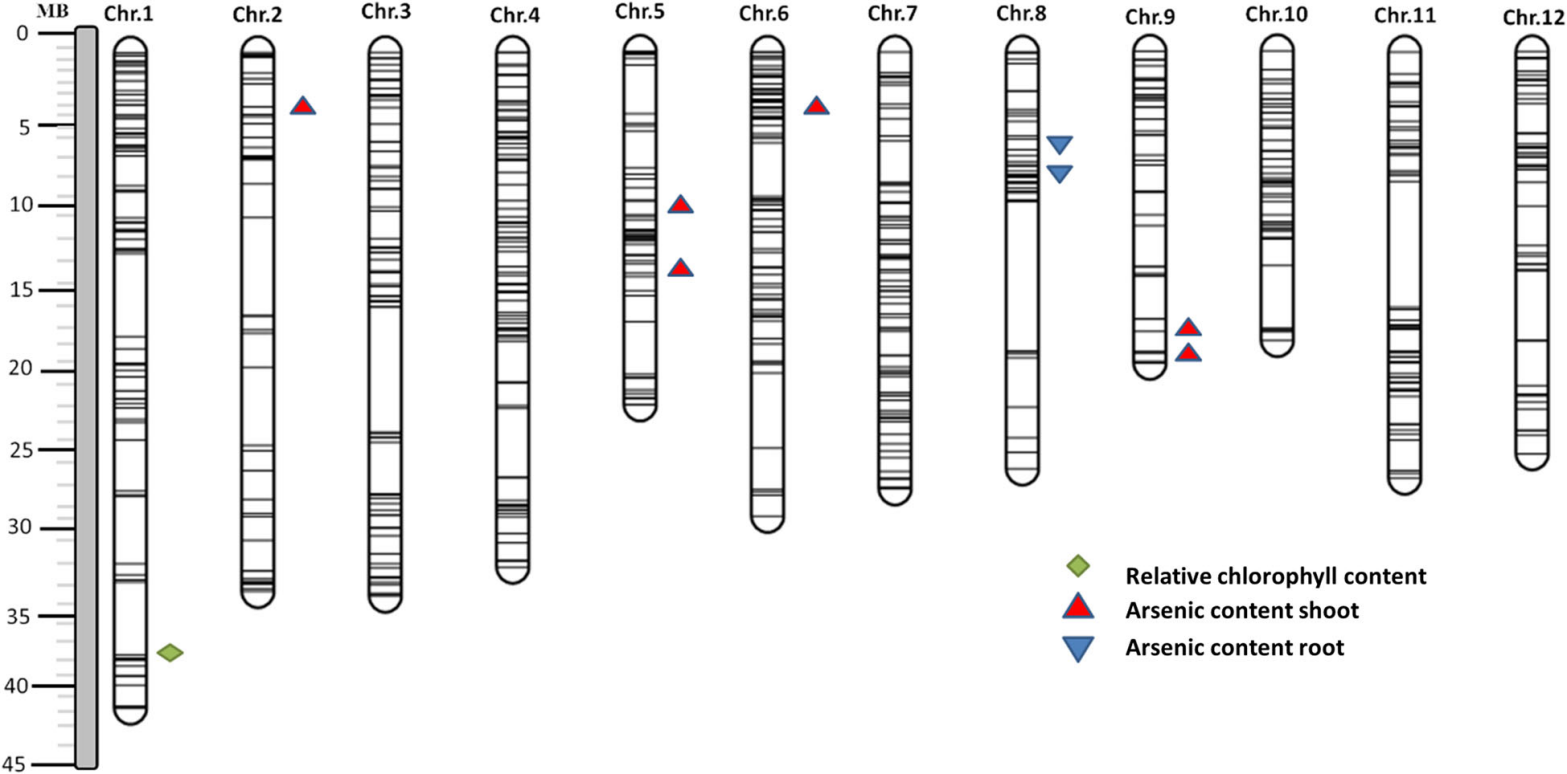
Chromosomal distribution of polymorphic single nucleotide polymorphisms (SNPs) and quantitative trait loci (QTLs) detected in this study. QTLs were located on the chromosome based on the physical position of the SNP marker

A customized 6 K SNP-Beadchip was used. It consists of 4606 high-quality SNP markers, which detected 1068 polymorphic sites between the parents and BC_1_F_6_ BRILs. A total of 704 polymorphic SNPs remained after pairwise comparison of SNPs between the parents for missing and heterozygous SNPs. These 704 SNPs were unevenly distributed across the genome, ranging from 84 SNPs on chromosome 4 to 41 on chromosome 9, with an average spacing of ∼524 kb between the SNPs, ranging from 393.9 kb on chromosome 7 to 797.4 kb on chromosome 2 (Additional file 2: Figure S1). There were 42 large gaps (> 2 Mb) across the genome in the distribution of SNPs generated by the 6 K SNP-array as a result of monomorphic SNP markers shared between parents in these regions. Gaps ranging from 4 Mb on chromosome 2 to 8 Mb on chromosome 11 were observed in the generated physical map of the rice genome using the 704 SNP marker data (Fig. 4). These 704 filtered SNPs were used to analyze the association between markers and As-related traits.

### Identification of QTLs for as-related traits

As tolerance are a non-target trait that was not considered during the development of the BRILs population used in this study. Therefore, the selected BRILs population could be considered as a random segregation population for mapping As related QTLs, assuming As tolerance was not correlated with those selected traits used in population development (yield under irrigated, rainfed, drought and low input). In total, 40 SNPs showed significant marker-trait association for the As-related traits (Table 2). Nine QTLs were defined by assuming that closely linked significant markers are in the QTL region. Among the nine QTLs, six were identified for As content in shoots, two were identified for As content in roots, and one tolerant QTL was identified for relative chlorophyll content. QTLs for As content in shoots (*qAsS2, qAsS5.1, qAsS5.2, qAsS6, qAsS9.1,* and *qAsS9.2*) were mapped on chromosomes 2, 5, 6, and 9, respectively. QTLs for As content in roots (*qAsR8.1* and *qAsR8.2)* were mapped on chromosome 8 and relative chlorophyll content QTL *qRChlo1* was mapped on chromosome 1 (Fig. 4). All the QTLs detected (*P* < 0.01) had a –log *p*(F) ≥3.3 and phenotypic variance explained by the QTLs ranged from 8.6% to 12.6%, with QTLs *qAsS5.2, qAsS6,* and *qAsR8.1* explaining major phenotypic variance of more than 10% and QTLs *qRChlo1, qAsR8.2, qAsS2, qAsS5.1, qAsS9.1,* and *qAsS9.2* explaining minor phenotypic variance of less than 10% (Table 2).

**Table 2:** QTLs associated with As-related traits after 18 days of 10 ppm As stress in WTR1× Hao-an-nong population by single marker regression

QTL^a^	Trait	Chr	Position^b^	Associated marker^c^	-log p(F)^d^	R^2^ (%)^e^	Additive effect^f^	Tolerance allele^g^
*qRChlo1*	Relative chlorophyll content	1	39.2829	66SNP_1_39282883	3.7781	8.6216	4.0676	WTR1
		1	39.3692	67SNP_1_39369209	3.7781	8.6216	4.0676	WTR1
		1	39.4208	68SNP_1_39420824	3.7781	8.6216	4.0676	WTR1
*qAsR8.1*	Arsenic content root	8	6.0577	487SNP_8_6057678	4.652	10.5069	− 11.7883	WTR1
*qAsR8.2*	Arsenic content root	8	7.854	492SNP_8_7854002	3.821	8.7155	−10.6686	WTR1
*qAsS*2	Arsenic content shoot	2	4.3429	88SNP_2_4342883	4.3275	9.8119	−1.1264	WTR1
		2	4.9307	89SNP_2_4930742	4.1432	9.4147	−1.1015	WTR1
		2	5.8303	90SNP_2_5830265	4.0335	9.1768	−1.0944	WTR1
		2	6.4799	91SNP_2_6479920	4.1741	9.4812	−1.1054	WTR1
		2	7.0233	92SNP_2_7023295	4.1741	9.4812	−1.1054	WTR1
		2	7.0767	93SNP_2_7076671	4.332	9.8214	−1.1248	WTR1
		2	7.1037	94SNP_2_7103684	4.332	9.8214	−1.1248	WTR1
		2	7.1708	95SNP_2_7170842	4.332	9.8214	−1.1248	WTR1
		2	7.2388	96SNP_2_7238793	4.332	9.8214	−1.1248	WTR1
		2	7.2775	97SNP_2_7277487	4.1741	9.4812	−1.1054	WTR1
*qAsS*5.1	Arsenic content shoot	5	10.8863	287SNP_5_10886331	3.7203	8.4964	−1.0489	WTR1
		5	13.3255	288SNP_5_13325546	3.7203	8.4964	−1.0489	WTR1
		5	13.7687	289SNP_5_13768744	3.7203	8.4964	−1.0489	WTR1
		5	14.0797	290SNP_5_14079677	3.7203	8.4964	−1.0489	WTR1
		5	14.644	291SNP_5_14643984	4.1216	9.3682	−1.1564	WTR1
*qAsS5.2*	Arsenic content shoot	5	15.4693	292SNP_5_15469279	4.0303	9.1701	−1.0871	WTR1
		5	15.556	293SNP_5_15556017	4.0303	9.1700	−1.0871	WTR1
		5	16.4598	294SNP_5_16459802	4.0303	9.1701	−1.0871	WTR1
		5	16.5851	295SNP_5_16585060	4.0303	9.1700	−1.0871	WTR1
		5	16.8086	296SNP_5_16808642	5.6649	12.6433	−1.3646	WTR1
*qAsS6*	Arsenic content shoot	6	0.4008	329SNP_6_400753	4.1072	9.3372	1.3763	Hao-an-nong
		6	0.6469	330SNP_6_646915	4.4046	9.9782	1.4682	Hao-an-nong
		6	0.8342	332SNP_6_834170	4.1072	9.3372	1.3763	Hao-an-nong
		6	1.5219	335SNP_6_1521855	3.5808	8.1922	1.3782	Hao-an-nong
		6	1.768	336SNP_6_1768006	5.323	11.929	1.4867	Hao-an-nong
		6	1.9284	337SNP_6_1928403	4.0138	9.1355	1.2804	Hao-an-nong
		6	1.9783	338SNP_6_1978288	4.0138	9.1355	1.2804	Hao-an-nong
		6	2.0107	339SNP_6_2010737	4.5116	10.208	1.3335	Hao-an-nong
		6	2.0256	340SNP_6_2025629	4.5116	10.208	1.3335	Hao-an-nong
*qAsS9.1*	Arsenic content shoot	9	18.3666	551SNP_9_18366555	3.884	8.8531	−1.085	WTR1
		9	18.3891	552SNP_9_18389054	3.884	8.8531	−1.085	WTR1
		9	19.2081	553SNP_9_19208050	3.7654	8.5948	−1.0579	WTR1
*qAsS9.2*	Arsenic content shoot	9	20.587	555SNP_9_20587039	3.9802	9.0617	−1.0806	WTR1
		9	21.2154	557SNP_9_21215424	3.6667	8.379	−1.0403	WTR1
		9	21.3489	558SNP_9_21348882	4.368	9.8987	−1.1294	WTR1

^a^Closely linked markers are assumed as the same QTL. ^b^Physical position of markers on chromosomes

^c^Marker associated with QTL. ^d^F-statistical analysis indicates association between markers and trait

^e^Proportion of phenotypic variance explained. ^f^Positive/negative values indicate that WTR1/ Hao-an-nong can increase trait values

^g^Tolerance allele provided by parental line

### Selection of candidate genes associated with as-related traits

**Fig. 5:**
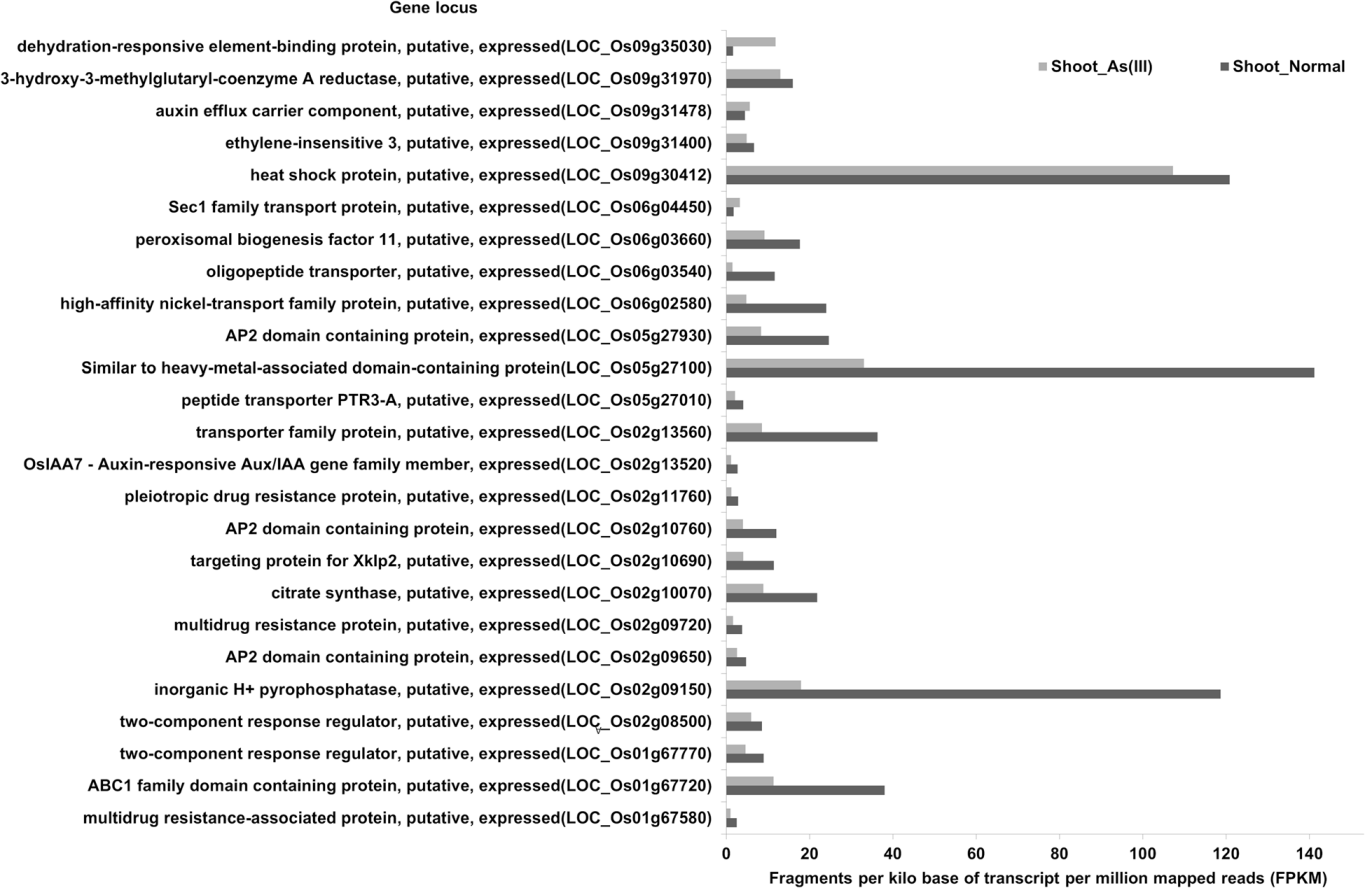
Transcriptional regulation of genes contained in identified QTL regions. Only genes containing non-synonymous SNPs showing significant expression response under As^(III)^ stress are plotted. Gene expression data adopted from Yu et al. (2012)

In the confidence interval of the identified QTLs, 35,767 and 440 SNPs were identified between the parents in the Rice SNP-Seek Database and tGBS® dataset, respectively. Among those identified SNPs, 35% of the SNPs showed polymorphism between the parents in 1309 genes, and most of these polymorphisms (93.9%) were synonymous mutations, with non-synonymous mutations (6.1%) between the parents in 676 genes (Table 3 and Additional file 3: Table S2). Among those 676 genes, 35 genes were associated with metal transport, response, and metal homeostasis and thus considered as the most likely candidate genes (Additional file 4: Table S3). Expression data of these genes were retrieved from a study by Yu et al. (2012) using the web application Rice Expression Database (Xia et al. 2017). Twenty-five genes out of the 35 most likely candidate genes showed expression responses to the sodium arsenite (As^III^) stress treatment according to expression data (Yu et al. 2012). Among the 25 genes showing expression responses, 3 genes were upregulated, and 22 showed significant down-regulation (Fig. 5).

**Table 3:** SNPs identified in the QTL regions using tGBS and 3 K RGP

QTLs	Chromosome	No. of SNPs in tGBS	No. of SNPs in 3 K RGP	Polymorphic SNPs	Non-synonymous SNPs	Total no. of genes	Genes with non-synonymous SNPs
*qRChlo1*	1	4	479	202	63	20	20
*qAsR8.1*	8	34	2761	757	148	47	27
*qAsR8.2*	8	65	3070	1110	179	61	42
*qAsS2*	2	79	11,705	4542	821	382	225
*qAsS5.1*	5	46	2346	677	108	94	45
*qAsS5.2*	5	103	2166	976	192	113	67
*qAsS6*	6	86	6789	1932	357	240	98
*qAsS9.1*	9	11	6154	2214	309	326	141
*qAsS9.2*	9	12	297	142	16	26	11
Total		440	35,767	12,552	2193	1309	676

## Discussion

In this study, we exposed two parental lines (WTR1/ Hao-an-nong) and 194 BC_1_F_6_ lines to a concentration of 10 ppm As^(III)^ at the seedling stage for 18 days in a hydroponic-based experimental setup. Similar concentrations frequently occur in the topsoil of the rice-growing regions of Bangladesh and West Bengal of India (Abedin et al. 2002a; Zhang et al. 2008). When exposed to 10 ppm of As^(III)^, a wide range of morpho-physiological changes occurred in the plants, leading to inhibited plant growth with a rapid decline in biomass and reduced chlorophyll content when compared to the control (Fig. 1) (Wu et al. 2011; Pandey et al. 2015). In our current experiment, the distribution of As showed similar patterns (root>shoot), in agreement with the previously reported genotype-dependent As accumulation and distribution, as the results suggested that the *indica* recipient parent (WTR1) accumulated less As in shoots than its *japonica* donor parent (Hao-an-nong) (Rahman et al. 2007; Bhattacharya et al. 2010; Wu et al. 2011). Correlation among the traits revealed that plant performance under As stress depended on As concentration in the tissue, with increased uptake in shoots reducing the plant’s overall performance. Arsenic translocation to shoots was positively correlated with As content in roots, which indicates that avoiding As translocation to shoot tissue will, in turn, increase plant performance by an As avoidance mechanism. In one earlier study, a doubled-haploid (DH) population derived from cross CJ06 × TN1 (*japonica* × *indica*) cultivars was used to evaluate the toxic effect of 10 ppm As^(III)^ in a soil-based controlled greenhouse experiment. The correlation in that study revealed results similar to those of the current study, clearly indicating that the performance of plants under As stress is inversely related to the As content in shoots (Fig. 3) (Zhang et al. 2008). Arsenic toxicity traits observed in the rice population in our experimental conditions signify that As tolerance and accumulation in rice seedlings is a quantitatively inherited trait controlled by multiple genes. Overall, selecting lines with the shoot avoidance mechanism would be appropriate for As-contaminated regions since farmers in the major rice-growing regions of India and Bangladesh use rice straw to feed farm cattle (Azizur Rahman et al. 2008). Some of the lines from the population showing high tolerance and low accumulation of As in shoots could be used in breeding programs aiming to identify genes that could increase As tolerance with low accumulation (Additional file 1: Table S1) (Tuli et al. 2010).

When using a 6 K SNP-array for genotyping, the average number of polymorphic markers derived across diverse germplasm was quite high (Thomson et al. 2017). However, only 1068 SNP sites were identified in this study, with 704 polymorphic sites remaining after removing the monomorphic sites. As the population used in the current study was advanced using the backcross breeding approach, only a single fragment or a small number of genomic introgression fragments from a donor parent were retrieved in the population, leading to the lower number of polymorphic sites present in the near-isogenic lines (Li and Ali 2017; Ali et al. 2018a). QTL mapping was performed using 704 SNP markers, and a total of 9 tightly linked QTLs associated with As-related traits were mapped using marker-trait association. The putative QTL regions detected for As-related traits in our study were compared with previous reports based on the physical positions of the associated markers in the Nipponbare genome (International Rice Genome Sequencing Project, http://rgp.dna.affrc.go.jp/IRGSP/). Associated SNPs mapped on chromosomes 2, 6, and 8 were consistent with previous studies for As content QTLs in rice (Dasgupta et al. 2004; Zhang et al. 2008; Chen et al. 2013). On the other hand, the QTLs mapped on chromosomes 1, 5, and 9 were reported for the first time in the As^(III)^-toxic treatment. Detection of multiple QTLs for traits in stable bi-parental populations illustrates the complexity of As^(III)^-induced traits (Pascual et al. 2016).

We employed a whole-genome sequencing approach of parents to narrow down the number of genes in the QTL intervals since QTLs mapped on the bi-parental populations are the only loci involved in a gene pool limited to the founder parents of the populations studied (Xu et al. 2017). By analyzing non-synonymous mutations in genes between the parental sequence in the QTL interval, we were able to narrow down the number of candidate genes from 1306 to 676. In order to further narrow down the number of candidate genes, we analyzed transcriptional regulation in response to As^(III)^-toxic conditions based on the data adopted from a previous expression study (Xia et al. 2017) (Fig. 5). Transcriptional analysis revealed As^(III)^ stress-responsive gene expression in the identified QTL regions, loci *LOC_Os05g27100, LOC_Os02g09150,* and *LOC_Os06g03540* present in the QTL regions *qAsS5.2, qAsS2,* and *qAsS6,* respectively, which showed significant down-regulation in gene expression after 24-h As^(III)^ treatment. All three highly down-regulated gene expressions carry one non-synonymous mutation between the parents in the coding region of the gene. Gene loci *LOC_Os09g35030* and *LOC_Os01g67580* present in the QTL regions *qAsS9.1* and *qRChlo1*, respectively, showed significant up-regulation after 24-h As^(III)^ treatment. Gene locus *LOC_Os01g67580* was highly polymorphic with seven non-synonymous mutations present in the coding region of the gene and locus *LOC_Os09g35030* carried one non-synonymous mutation between the parents in the gene-coding region (Additional file 3: Table S2). Overall, the whole-genome sequence of parents and gene expression analysis proved to be an effective strategy to narrow down the candidate genes in the QTL intervals.

Plants have evolved versatile detoxification systems to counter the wide variety of phytotoxic compounds present in the natural environment (Coleman et al. 1997; Shamsi et al. 2008). In the region 39.28–39.42 Mb on chromosome 1, QTL *qRChlo1* was located. Of the four candidate genes, the most plausible one was *LOC_Os01g67580* (*OsMRP2*), which encodes for a multidrug resistance-associated protein (MRP, ABCC1), a subfamily of ABC transporters. Members of the MRP ATP-binding cassette transporters were originally proposed to be primarily involved in the vacuolar sequestration of potentially toxic metabolites. MRPs exist in all organisms and are known to play key roles in the efflux of xenobiotic compounds (Paumi et al. 2009). In one study involving *Arabidopsis thaliana* double knockout mutants of ABCC- type transporters (AtABCC1 and AtABCC2), the mutant plants exhibited a very low residual As^(III)^–PC2 transport activity (Song et al. 2010). In another study, involving the same double knockout mutant lines (AtABCC1 and AtABCC2), tolerance of mercury and cadmium toxicity was conferred by sequestering in the large vacuole (Park et al. 2012). Another study, involving an AtABCC3 transporter mutant in *A. thaliana,* also confirmed increasing sensitivity to cadmium toxicity in the seedlings (Brunetti et al. 2015). The gene *OsMRP2*, an ABCC1 transporter identified in the QTL region of *qRChlo1,* may possibly be involved in As transport and partitioning processes such as vacuolar sequestration of toxic metabolites by the action of ATP-driven efflux pumps (Klein et al. 2006; Jeandroz and Lamotte 2017).

QTLs associated with shoot As content included genes of complex physiological and molecular networks, ranging from metal transporters, sequestration and partitioning of As, and synthesis of detoxification proteins and cheaters that promote shoot-to-root mobility of As (Tripathi et al. 2013; Pandey et al. 2015). In the region of 10.88–14.64 Mb on chromosome 5, QTL *qAsS*5.1 was located. Of the three candidate genes, the most likely one was *LOC_Os05g27100,* annotated as heavy metal-associated domain-containing protein (HMA). HMAs are metallochaperones that contain a metal binding domain for safe transport of metallic ions inside and outside of the cell, primarily involved in detoxification mechanism (De Abreu-Neto et al. 2013). In rice, gene *OsHMA9* is a metal efflux HMA protein known to play a key role in metal homeostasis of copper, zinc, lead, and cadmium (Lee et al. 2007). In the case of As toxicity in rice, the role of HMA is not yet defined. In the region of 4.34–7.22 Mb on chromosome 2, QTL *qAsS*2 was located. Of the 13 candidate genes, the most likely one was *LOC_Os02g09150*, which encodes for inorganic H+ vacuolar pyrophosphates (V-PPase). Plant V-PPase is a specific class of multi-subunit pumps that play an essential role in the productivity of higher plants, generation of proton gradients across tonoplast endomembranes, and their ability to buffer changes in the concentrations of essential and toxic ions (Bak et al. 2013). A study involving *A. thaliana* double knockout mutant lines of V-PPase (vha-a2 and vha-a3) showed reduced zinc tolerance with no response to salt stress (Krebs et al. 2010). Another study involving the same double knockout mutant lines (vha-a2 and vha-a3) displayed functional regulation of intracellular ion homeostasis by ion compartmentalization of calcium ion (Tang et al. 2012). However, their functional relation and relative contributions to ion storage and detoxification are unclear in rice. In the region of 0.40–2.02 Mb on chromosome 6, QTL *qAsS*2 was located. Of the six candidate genes, the most likely one was *LOC_Os06g03540* (*OsOPT2*) annotated as oligopeptide transporter family (OPT), which plays an important and diverse role in plant growth and development. It is widely accepted that OPTs are proton-coupled symporters that translocate their substrates in the cytosolic direction with the possible function of long-distance metal distribution, nitrogen mobilization, heavy metal sequestration, and glutathione transport (Lubkowitz 2011). Studies suggest that 16 OPT gene motifs are distributed in the rice genome, and *OsOPT2* showed transcriptional accumulation in all tissues, with varying amounts under normal conditions and significantly down-regulated under salinity and cold stress and up-regulated under drought stress, suggesting that *OsOPT2* may be involved in diverse plant regulation functions (Liu et al. 2012b). However, *OsOPT2* was not functionally characterized in rice.

In most cases, including root-related metal concentration traits, excess mineral attached to the root surface may interfere with the measurement. This measurement is not considered as a good-quality trait for understanding mineral uptake and metabolism in rice (Zhang et al. 2008). Two QTLs identified for root As content, *qAsR*8.1 (5.84–6.28 Mb) and *qAsR*8.2 (7.33–8.38 Mb), were located 1 Mb apart from each other on chromosome 8, and co-localized with a QTL previously reported for As content in brown rice (Zhang et al. 2008). Within the region was harbored the well-studied rice gene (*OsZIP4*) responsible for zinc transport and distribution in rice (Ishimaru et al. 2005, 2007). Moreover, genes present in the root QTL intervals for As content did not show any response in the gene expression analysis.

Reducing the levels of the carcinogenic As in rice is a major health goal. To the best of our knowledge, this study is the first to use the breeding population to map QTLs associated with As toxicity stress in rice. For the QTLs identified in this study to make an impact on adaptive rice breeding, it is necessary to evaluate both parents (WTR1 and Hao-an-nong) and BRILs under high As contaminated soil to understand the As tolerance and accumulation. Further, low As accumulating BRILs (AsG187 and ASG189) identified through this study needs to be tested under high As contaminated soil for As content in mature brown rice and straw before any varietal nomination in the contaminated region. For the development of As-safe rice, the candidate genes nominated in this study need to be verified and functionally characterized.

## Conclusions

This study provides novel insights into the genetic basis of As^III^ toxicity tolerance and accumulation in rice. Through marker-trait association analysis, a total of nine QTLs were identified on the different chromosomes in rice. Genetically dissecting As toxicity tolerance and accumulation is complex because it is a quantitatively inherited trait controlled by multiple genes. This study also demonstrates that using the whole-genome sequencing of parents and expression analysis provides useful information about the candidate genes present in the QTL intervals, and a total of 25 promising candidate genes for 9 QTL regions were determined. The candidate genes associated with As toxicity tolerance and accumulation identified in this study provide a valuable basis for future functional gene characterization and improvement of rice varieties for As-contaminated ecosystems.

## Methods

### Plant materials and phenotypic screening

A population of 230 BC_1_F_6_ backcross recombinant inbred lines (BRILs) derived from a cross between *indica* line WTR1 and *japonica* cultivar Hao-an-nong was produced using single seed descent at the International Rice Research Institute (IRRI), Los Baños, Philippines. For detailed information regarding the population development, see (Jewel et al. 2019). As tolerance and accumulation was a non-target trait that was not considered during the development of the BRILs population which was used in this study. Thus, the selected BRILs population could be considered as a random segregating population for mapping As related QTLs, assuming As tolerance and accumulation was not correlated with those selected traits used in population development (yield under irrigated, rainfed, drought and low input condition). For the hydroponic screening experiment, 194 lines from the developed population were used, and the experiment was carried out in a controlled phytotron glasshouse of IRRI. Optimum rice-growing conditions were maintained throughout the experiment: 29/21 °C (day/night), 70% relative humidity, and natural light. Seeds of the 194 lines were oven-dried for 5 days at 60 °C to break any residual seed dormancy and incubated at 30 °C for 48 h for germination. One seedling per line was transferred per hole with 1 cm diameter on a Styrofoam seedling float with size of 28 × 32 × 1.25 cm having 100 holes (10 × 10) with a nylon net bottom-fixed in a dark plastic tray containing 8 L of full-strength Yoshida nutrient solution (Jodal et al. 1975; Wu et al. 2017) Throughout the experiment, pH was maintained in a range of 5.1 to 5.4 in the nutrient solution. On the seventh day, 10 ppm As was supplied in the form of sodium-(meta)-arsenite (As^III^) (AsNaO_2_, Sigma-Aldrich, MO, USA). A control treatment without As was maintained throughout the experiment. Plants were grown in As-toxic conditions for 18 days, and nutrient solutions were renewed once every 7 days. The experiment was set up as a complete randomized design with three independent replicates, and five repeats per line in each replicate, leading to a total of 59 hydroponic tanks, each accommodating up to 100 seedlings. To maintain uniform As concentration across the different hydroponic tanks, nutrient solutions were prepared in a single tank having 1000 L capacity and non-segregating parents (WTR1 and Hao-an-nong) were grown as checks in each hydroponic tanks. Any positional effect within the phytotron glasshouse was minimized by altering the position of hydroponic tanks every second day.

### Chlorophyll concentration and seedling growth parameters

Leaf yellowing and senescence were observed in plants on the 12th day post-As treatment (PAsT). To compare the differences among the lines, the relative chlorophyll concentrations were measured non-destructively from the base, middle, and tip of the top leaves of each individual plant, and the average values were expressed as SPAD units (SPAD-502 chlorophyll meter, Minolta Camera Co., Ltd., Japan) as the indicator of leaf senescence caused by toxic As treatment. Plants’ responses to the As treatment were evident after 18 days PAsT. Changes in the shoot and root length response to the As treatment were measured for each entry at 18 days PAsT. Shoot length was measured from the base of the plant to the tip of the longest leaf, while root length was measured from the base of the plant to the root tip. Three plants per entry per replicate were rinsed with deionized water to remove culture solution sticking to the surface of the root and then oven-dried for 3 days at 70 °C to remove moisture. Before sample processing for As content analysis, dry biomass was recorded.

### Sample preparation and analysis of as concentration

Arsenic content in roots and shoots was determined for parents and BRILs grown in As-toxic conditions at 18 days PAsT. Dried samples were thoroughly homogenized by an ultracentrifugal mill (ZM100, Retsch, Haan, Germany) modified with a tungsten blade to avoid any cross-contamination. Samples ground to 0.5 g were added to a closed vessel digester and 5 mL of high-purity 69% concentrated nitric acid (HNO_3_) followed by 2 mL hydrogen peroxide (H_2_O_2_), and 1 mL of deionized water were added and predigested overnight in the fume hood (Amaral et al. 2013). The following day, the samples were digested using a heating block at 150–155 °C for 3 h under the fume hood. The digested tissue was diluted to a final volume of 25 mL using deionized water and total As was determined using Graphite Furnace Atomic Absorption Spectrophotometry (GFAAS-7000F, SHIMADZU Corporation, Kyoto, Japan) (Hirohashi 2001). A deuterium lamp background correction with high-performance As hollow cathode lamps (193.7 nm) was used to quantify the As content in the standards and samples. An aliquot of 20 μL of the digested sample and 10 *μ*L palladium (100 ppm) as matrix modifier were injected into the pyrol-coated graphite tube with the aid of an autosampler unit. A seven-stage furnace cycle program was adopted with atomization temperature of 2200 °C at the sixth stage used as a standard parameter to determine As content in the sample and expressed in milligrams per kilogram (mg kg^− 1^).

### DNA extraction and genotyping

Genomic DNA was extracted from the seedlings of 194 BRILs and two parents (WTR1 and Hao-an-nong) using DNeasy Plant Mini Kit following the manufacturer’s protocol (QIAGEN, Germantown, MD, USA). High-throughput SNP genotyping was carried out using a custom-design Illumina Infinium rice 6 K SNP-array containing 4606 SNPs covering all 12 rice chromosomes in the Genotyping Services Laboratory facility at IRRI (Thomson 2014; Thomson et al. 2017). A rice 6 K-SNP chip was scanned using an Illumina bead array reader, and automatic allele calling was achieved using Illumina Genome Studio data analysis software (V2010.1. Illumina Inc.) (Thomson et al. 2017). Scoring of alleles in the BRILs at each SNP locus was carried out by comparing parental alleles at the respective SNP locus as co-dominant markers.

### Statistical analysis and QTL mapping

Two-way ANOVA was conducted before obtaining the relative phenotypic trait values to observe the effects of lines, treatment (control and As treatment), and line-treatment interaction for different measured traits. Multiple comparison analysis was performed on parents following the least significant difference (LSD) post hoc test. Relative phenotypic value of morphological traits was determined for each line, that is, the phenotypic value obtained from the As treatment divided by the control value (Dasgupta et al. 2004; Shrestha et al. 2018). Pair-wise Pearson‘s correlation analysis was carried out among the As-related traits, in which *P-* value was two-tailed with two significant levels using *P* = 0.05 and *P* = 0.01, and a heat map was generated using *corrplot* package in R studio. All the statistical analyses were conducted using PBTools package of R (R Core Team 2015). For QTL mapping in the WTR1 × Hao-an-nong backcross breeding population, a physical map consisting of 704 SNP markers covering all 12 chromosomes based on the physical locations of the markers was constructed. QTL mapping was performed by single marker regression analysis using the function single marker analysis (SMA) of iciMapping program v.4.0 (www.isbreeding.net/software/?type=detail&id=18). The threshold (−log *p*(F) ≥3.3) to declare significant association was set based on a permutation test (1000 permutations, *P* = 0.01) for each trait (Crump and Llewellyn-Thomas 2011). Tightly linked SNP markers showing significant associations were assumed as the same QTL and named following the standard protocol (McCouch et al. 1997).

### Candidate gene identification

For the identified QTLs governing As-related traits, the gene models located in the QTL intervals were searched from the MSU Rice Genome Annotation Database (Ouyang et al. 2007). In the identified QTL regions, loci showing non-synonymous polymorphism between the parents were considered for nomination as candidate genes (Pang et al. 2017). Polymorphisms between parental lines in QTL regions were retrieved from the Rice SNP-Seek Database (http://snp-seek.irri.org) (Alexandrov et al. 2015). Additional genotype information on parents was obtained from the earlier Tunable Genotyping-By-Sequencing (tGBS®) project using 10 ion proton runs, resulting in 794,297 polymorphic SNPs (10.7910/DVN/RRXCR3) (Ali et al. 2018a). Raw sequence reads of parents obtained from the tGBS® project were aligned to the public reference rice genome Osativa_204_v7.0.fa, downloaded from the Phytozome website (https://phytozome.jgi.doe.gov/pz/portal.html#!info?alias=Org_%20Osativa). By using the Genomic Short-read Nucleotide Alignment program (GSNAP) (http://research-pub.gene.com/gmap/), short reads were confidently mapped to the best location in the reference genome by allowing ≤2 mismatches every 36 base pairs (bp), and fewer than 5 bases for every 75 bp as tails were used as criteria. Aligned reads of the parents were compared with the reference genome sequence for polymorphism sights and compared among the parents to obtain SNP information (Wu and Nacu 2010; Ott et al. 2017). Then, we selected gene models in the QTL intervals showing non-synonymous polymorphism associated with metal homeostasis as our most likely candidate genes. Expression profiles of the most likely candidates in the identified QTL regions were extracted from a previously published study in which rice cultivar Nipponbare (*Oryza sativa L. subsp*. *japonica*) had been exposed to sodium arsenite (As^III^) stress for 24 h in the seedling stage (Yu et al. 2012). Data from that expression profile experiment were publicly available (Xu et al. 2018) (Xia et al. 2017).

## Additional files

Additional file 1: **Table S1.** Mean relative phenotypic values of traits measured under As stress. (XLSX 32 kb)

Additional file 2: **Figure S1.** Distribution of SNPs on the chromosome. (PNG 49 kb)

Additional file 3: **Table S2.** Total number of genes and SNPs identified in the candidate region. (XLSX 110 kb)

Additional file 4: **Table S3.** Candidate genes present in the QTL intervals. (XLSX 12 kb)

## Data Availability

The comprehensive collected data supporting the conclusions of this review article are provided as figures, tables, and supplementary tables.

## Abbreviations

As: Arsenic
AWD: Alternate wetting drying
BRILs: Backcross recombinant inbred lines
DSR: Direct seeded rice
GSR: Green super rice
PAsT: Post arsenic treatment
QTL: Quantitative trait locus
SNP: Single nucleotide polymorphism
tGBS: Tunable Genotyping by Sequencing
