# Supplementary figures and images for: Mapping of genomic regions associated with arsenic toxicity stress in a backcross breeding populations of rice (Oryza sativa L.)
Source: Rice (N Y). 2019 Aug 9;12:61. doi: 10.1186/s12284-019-0321-y (PMC6689042; doi:10.1186/s12284-019-0321-y)

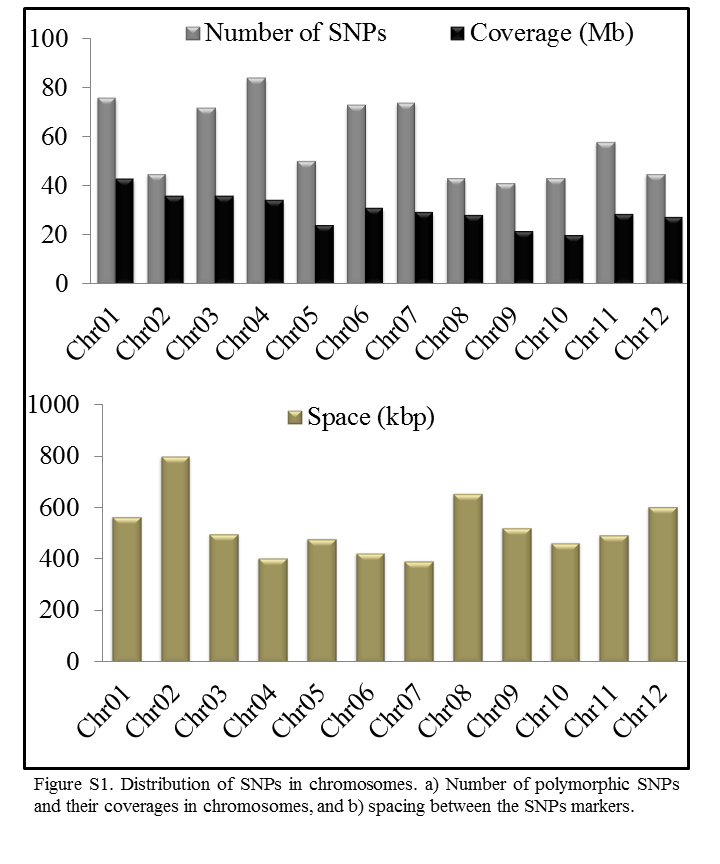

Supplement: Supplementary file 2 — Figure S1. Distribution of SNPs on the chromosome. (PNG 49 kb) [file 12284_2019_321_MOESM2_ESM.png]
